# Response of Submerged Macrophyte Communities to External and Internal Restoration Measures in North Temperate Shallow Lakes

**DOI:** 10.3389/fpls.2018.00194

**Published:** 2018-02-19

**Authors:** Sabine Hilt, Marta M. Alirangues Nuñez, Elisabeth S. Bakker, Irmgard Blindow, Thomas A. Davidson, Mikael Gillefalk, Lars-Anders Hansson, Jan H. Janse, Annette B. G. Janssen, Erik Jeppesen, Timm Kabus, Andrea Kelly, Jan Köhler, Torben L. Lauridsen, Wolf M. Mooij, Ruurd Noordhuis, Geoff Phillips, Jacqueline Rücker, Hans-Heinrich Schuster, Martin Søndergaard, Sven Teurlincx, Klaus van de Weyer, Ellen van Donk, Arno Waterstraat, Nigel Willby, Carl D. Sayer

**Affiliations:** ^1^Department of Ecosystem Research, Leibniz-Institute of Freshwater Ecology and Inland Fisheries, Berlin, Germany; ^2^Departmnet of Aquatic Ecology, Netherlands Institute of Ecology (NIOO-KNAW), Wageningen, Netherlands; ^3^Biological Station of Hiddensee, University of Greifswald, Greifswald, Germany; ^4^Department of Bioscience, Aarhus University, Silkeborg, Denmark; ^5^Department of Biology, Lund University, Lund, Sweden; ^6^Netherlands Environmental Assessment Agency (PBL), Den Haag, Netherlands; ^7^Water Systems and Global Change Group, Wageningen University and Research, Wageningen, Netherlands; ^8^Sino-Danish Centre for Education and Research, University of Chinese Academy of Sciences, Beijing, China; ^9^Institute of Applied Freshwater Ecology, Seddiner See, Germany; ^10^Broads Authority, Norwich, United Kingdom; ^11^Department of Aquatic Ecology and Water Quality Management, Wageningen University and Research, Wageningen, Netherlands; ^12^Deltares, Delft, Netherlands; ^13^Biological and Environmental Sciences, University of Stirling, Stirling, United Kingdom; ^14^Department of Freshwater Conservation, Brandenburg University of Technology Cottbus-Senftenberg, Senftenberg, Germany; ^15^Niedersächsischer Landesbetrieb für Wasserwirtschaft, Küsten- und Naturschutz, Sulingen, Germany; ^16^Lanaplan, Nettetal, Germany; ^17^Gesellschaft für Naturschutz und Landschaftsökologie, Kratzeburg, Germany; ^18^Department of Geography, Environmental Change Research Centre, University College London, London, United Kingdom

**Keywords:** aquatic plants, biomanipulation, eutrophication, lake restoration. nutrient load reduction, PCLake, plant traits, regime shift

## Abstract

Submerged macrophytes play a key role in north temperate shallow lakes by stabilizing clear-water conditions. Eutrophication has resulted in macrophyte loss and shifts to turbid conditions in many lakes. Considerable efforts have been devoted to shallow lake restoration in many countries, but long-term success depends on a stable recovery of submerged macrophytes. However, recovery patterns vary widely and remain to be fully understood. We hypothesize that reduced external nutrient loading leads to an intermediate recovery state with clear spring and turbid summer conditions similar to the pattern described for eutrophication. In contrast, lake internal restoration measures can result in transient clear-water conditions both in spring and summer and reversals to turbid conditions. Furthermore, we hypothesize that these contrasting restoration measures result in different macrophyte species composition, with added implications for seasonal dynamics due to differences in plant traits. To test these hypotheses, we analyzed data on water quality and submerged macrophytes from 49 north temperate shallow lakes that were in a turbid state and subjected to restoration measures. To study the dynamics of macrophytes during nutrient load reduction, we adapted the ecosystem model PCLake. Our survey and model simulations revealed the existence of an intermediate recovery state upon reduced external nutrient loading, characterized by spring clear-water phases and turbid summers, whereas internal lake restoration measures often resulted in clear-water conditions in spring and summer with returns to turbid conditions after some years. External and internal lake restoration measures resulted in different macrophyte communities. The intermediate recovery state following reduced nutrient loading is characterized by a few macrophyte species (mainly pondweeds) that can resist wave action allowing survival in shallow areas, germinate early in spring, have energy-rich vegetative propagules facilitating rapid initial growth and that can complete their life cycle by early summer. Later in the growing season these plants are, according to our simulations, outcompeted by periphyton, leading to late-summer phytoplankton blooms. Internal lake restoration measures often coincide with a rapid but transient colonization by hornworts, waterweeds or charophytes. Stable clear-water conditions and a diverse macrophyte flora only occurred decades after external nutrient load reduction or when measures were combined.

## Introduction

Shallow lakes are the most abundant freshwater ecosystems on earth (Verpoorter et al., 2014). In their pristine state, they are often characterized by abundant submerged vegetation which can stabilize clear-water conditions (Scheffer et al., 1993) and plays a key role in the functioning of the ecosystem (Hilt et al., 2017). Several mechanisms contribute to a positive feedback between macrophytes and clear water conditions. As a consequence, shallow lakes are resistant to increasing nutrient loading up to a critical threshold, above which their macrophytes collapse and the lakes shift into a turbid, phytoplankton-dominated state (Scheffer et al., 1993). In recent centuries, excessive nutrient loading has resulted in a loss of macrophytes and shift to this turbid state in many temperate shallow lakes (e.g., Körner, 2002; Phillips et al., 2016).

Sayer et al. (2010a) suggested a typical pattern of lake macrophyte loss, defining a so-called “crashing” state lying between the stable clear-water state featuring a diverse plant community and the final turbid state lacking in macrophytes. This crashing state is characterized by the occurrence of only a few macrophyte species that can complete their life cycle during clear-water conditions in spring and early summer while later in summer, cyanobacteria blooms often occur. Eventually, the remaining macrophyte stands are also lost and give way to year-round phytoplankton dominance (Sayer et al., 2010a,b, 2016). Under these conditions, several ecosystem functions and services deteriorate, including biodiversity support, nutrient retention, provision of water of drinking or swimming quality (Hilt et al., 2017).

Hence, considerable efforts and financial resources have been devoted to the restoration of shallow lakes in many countries in recent decades (Jeppesen et al., 2005). The success of lake restoration in the long-term depends critically on the stable recovery of submerged macrophytes (Hilt et al., 2006). However, the turbid state is stabilized by feedback mechanisms that can prevent macrophyte re-colonization even at reduced nutrient loading. In theory, only the reduction of nutrient levels below a critical threshold or a significant reduction in the abundance of planktivorous and benthivorous fish (e.g., by biomanipulation or natural fish kills) will lead to a recovery of clear-water conditions and a return of macrophytes (Scheffer et al., 1993). In practice, reductions in the external nutrient load to shallow lakes often fail to deliver macrophyte recovery (Jeppesen et al., 2005). Similarly, biomanipulation of the fish community in turbid shallow lakes has produced variable effects on macrophytes in shallow lakes (Hansson et al., 1998; Bergman et al., 1999; Søndergaard et al., 2008; Jeppesen et al., 2012; Bernes et al., 2015; Sayer et al., 2016). Overall, the response of macrophyte communities to different types of lake restoration measures remains to be fully understood (Jeppesen et al., 2005; Bakker et al., 2013).

**Figure 1:**
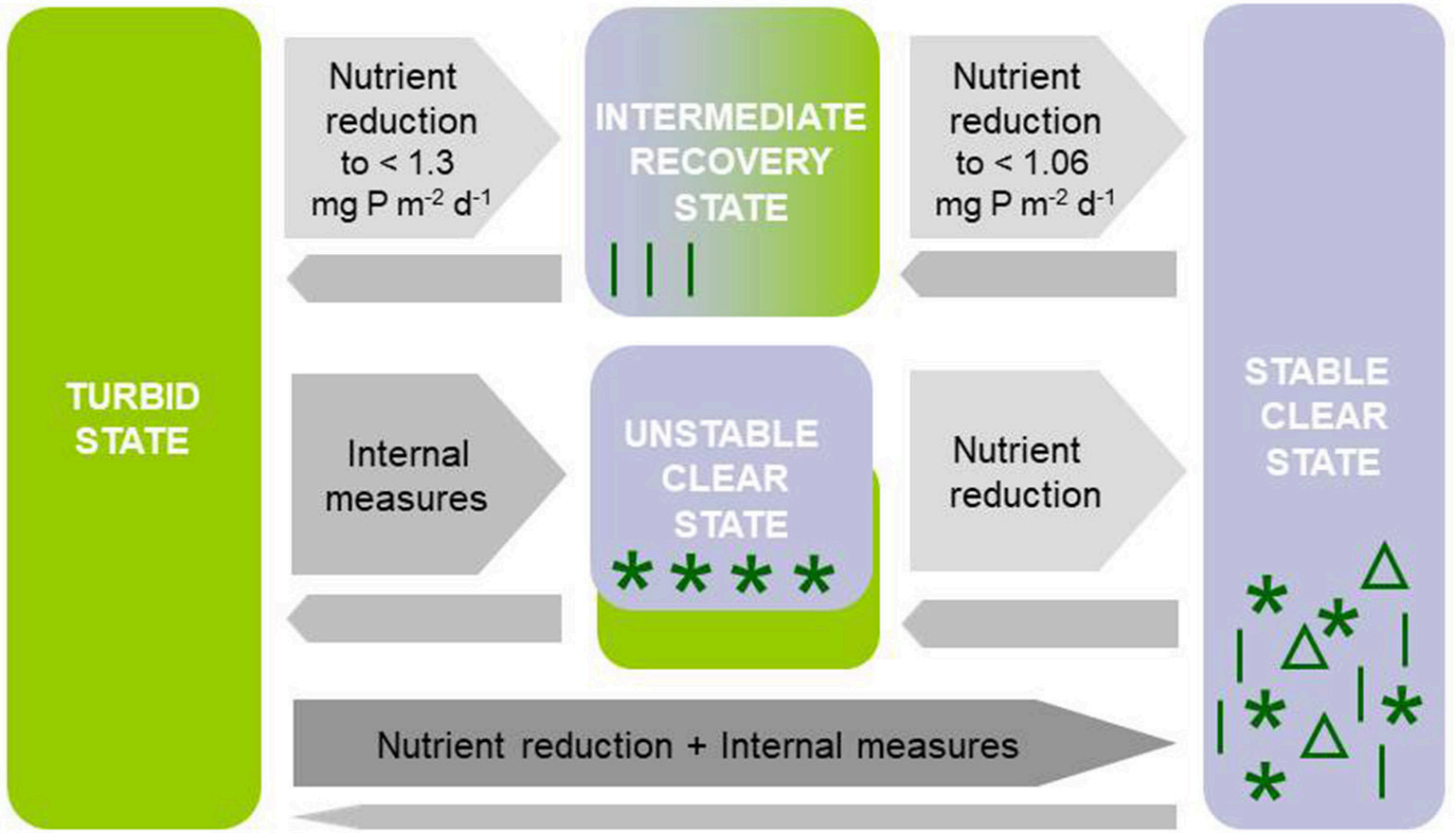
Response patterns of turbid north temperate shallow lakes to different restoration measures: (1) External restoration measures (reduction of external nutrient loading) are expected to lead to an intermediate recovery state with clear-water conditions in spring and turbid water in summer and specific macrophyte communities with short growth seasons and eventually stable clear conditions with a diverse macrophyte flora if nutrient loading is reduced sufficiently or additional internal measures are applied (reversed order as suggested for eutrophication by Sayer et al., 2010a,b). Thresholds in phosphorus (P) loading are based on simulations using PCLake (see **Figure 5**). (2) Lake-internal measures (biomanipulation, sediment suction dredging) leading to unstable clear-water conditions with specific macrophyte communities that may collapse resulting in a shift back to turbid conditions unless nutrient loading is reduced, or (3) a combination of external and internal restoration leading to stable clear-water conditions with an abundant and diverse macrophyte community.

We hypothesize that (1) external lake restoration measures leading to nutrient load reduction in turbid temperate shallow lakes result in macrophyte re-establishment in a reversed sequence to the one described by Sayer et al. (2010a,b) for advancing eutrophication. An intermediate recovery state should occur where the water is clear in spring but dominated by phytoplankton and thus turbid in late summer, until, eventually, seasonally stable conditions characterized by high water clarity in both spring and summer would dominate (Figure 1). In contrast, lake internal measures such as biomanipulation or phosphorus precipitation are expected to result in transient clear-water conditions in spring and summer if either zooplankton is sufficiently released from fish predation or internal phosphorus loading from sediments is reduced enough to control summer phytoplankton. Such conditions are supposed to occur only temporarily in the absence of additional external nutrient load reduction (Figure 1). We hypothesize that (2) these contrasting types of restoration measures result in different macrophyte community composition and seasonal patterns in plant abundance. Specific macrophyte communities with short growth seasons should dominate during the intermediate recovery state following external nutrient loading reduction, while species with longer growing season requirements are predicted to temporarily establish following lake-internal measures (Figure 1). The establishment of stable clear conditions with a diverse macrophyte community is thus assumed to require both, external and internal measures (Figure 1).

To test these hypotheses, we analyse existing data on the water quality and submerged macrophytes of 49 temperate shallow lakes that had deteriorated to a turbid state and subsequently were subject to either external or internal restoration measures or both. In addition, we use an adapted version of the ecosystem model PCLake (Janse et al., 2008) to simulate the response of water clarity and macrophyte biomass to external nutrient load reduction and to detect any thresholds in nutrient loading for macrophyte recovery. Traits of the typical macrophyte species found after external and internal lake restoration measures are compared to provide a mechanistic understanding of the observed re-colonization patterns.

## Materials and methods

### Literature and data search on macrophyte species recovery

We started our literature review with the 22 shallow lakes described in detail in the study by Jeppesen et al. (2005) on the response of lakes to reduced external nutrient loading. However, only two of these 22 lakes were turbid before the nutrient load reduction (Müggelsee, Veluwemeer) and both showed an increase in macrophyte coverage following the intervention. The rest showed no change, macrophyte declines or lacked suitable data (Jeppesen et al., 2005). Therefore, we searched for more examples in published and unpublished studies on lakes in Germany, The Netherlands, Denmark, southern Sweden and UK where shallow lakes are abundant and experience similar eutrophication problems and climatic conditions. We selected lakes that had lost most of their submerged macrophytes during a turbid phase and subsequently had been subjected to either external restoration via nutrient load reduction (summarized in Table 1), or internal restoration using biomanipulation or sediment dredging (Table 2). We did not carry out a full systematic review of available data, but instead focussed on known lakes within the research network of the authors where at least partial recovery through restoration measures was evident.

**Table 1:** The response of north temperate shallow lakes in Germany (DE), United Kingdom (UK), The Netherlands (NL), and Denmark (DK) to external nutrient load reduction (x: data on Secchi disk transparency and total phosphorus concentrations were available and used in Figure 2A).

**No**.	**Lake**	**Country**	**Size (ha)**	**Depth (max/mean) (m)**	**Measures of nutrient load reduction**	**Period of turbid conditions, major remaining macrophyte species and coverage (% lake area)**	**Period of intermediate recovery conditions, major macrophyte species and coverage (% lake area)**	**Period of stable clear conditions and macrophyte species**	**Data Figure 2A**	**References**
1	Großer Müggelsee	DE	750	8/4.9	Since 1989: improved wastewater treatment in catchment	1970–1989 Sparse stands of *P. pectinatus*	1990–2013 *P. pectinatus, P. perfoliatus, P. crispus, Z. palustris* ca. 5% (Figure 4)	2014-now *P. pectinatus, P. perfoliatus, Najas marina, E. nuttallii* ca. 25% (Figure 4)	x	Hilt et al., 2013, S. Hilt unpubl. data
2	Großer Wannsee		282	9.8/5.5	Nutrient load reduction in catchment		?-ongoing *P. pectinatus, P. crispus* <5%	not yet reached		Hilt and Grünert, 2008; Van de Weyer, 2011
3	Galen-becker See		590	1.8/0.8	Since 1972: reduction of P loading by treatment in upstream reservoir, since 2007 by regulation of whole water supply to lake	1995–2002	2003–2006 *P. pectinatus*	2008–now *Chara contraria, C. tomentosa, C. globularis, C. intermedia, Nitellopsis obtusa, N. marina f. intermedia, P. pectinatus, Z. palustris*	x	Waterstraat, 2008, unpubl. data
4	Dümmer		1350	4/1.1	Reduction of P load by wastewater treatment plants by 95%	1960–2011 *P. pectinatus* (rare)	2011–ongoing *P. pectinatus, P. crispus, Z. palustris, P. pusillus* 25-50%	not yet reached	x	Blüml et al., 2008; Schuster, unpubl. data
5	Schwielow-see		786	9.1/2.8	Nutrient load reduction in catchment area (River Havel)	?−2005	2006–now *P. pectinatus*	not yet reached	x	Kabus et al., 2007
6	Wuster-witzer See		172	9.2/3.4	Nutrient load reduction in catchment area	?−2005	2006–ongoing *P. pectinatus*	not yet reached	x	Kabus et al., 2007
7	Grimnitz-see		777	10.3/4.5	Since 1994: sewage treatment plant in operation	1970–1990 *P. pectinatus*	1991–? *P. pectinatus*	?–now *2008: C. contraria, N. obtusa, P. pectinatus, P. perfoliatus, C. demersum, L. trisulca 2001: C. contraria, C. globularis, N. obtusa, P. pectinatus*	x	Mauersberger and Mauersberger, 1996; Gervais et al., 1999; Kabus and Mauersberger, 2011
8	Wardersee		357	10.8/3.7	Wastewater treatment plants	?	1996–2006 *P. pectinatus, P. perfoliatus, P. crispus, Z. palustris*	?	x	Landesamt für Natur und Umwelt des Landes Schleswig-Holstein, 1997; Heinzel and Martin, 2006
9	Hemmels-dorfer See		450	6 (northern part)	1998: P load reduction	?-1978-?	?-2006-? *P. pectinatus, Z.palustris, P. perfoliatus, R. circinatus*	?		Heinzel and Martin, 2006
10	Großer Varchen-tiner See		182	1.7/?	Lowered nutrient input from agricultural catchment since 1990	?–?	?-2012-? *P. pectinatus, M. spicatum* 90%	2016 *M. spicatum, Najas marina* ssp. *intermedia*	x	Kabus unpubl. data
11	Großer Dambecker See		94	2.1/0.8	Lowered nutrient input from agricultural catchment since 1990	?- 2007 few *P. pectinatus*	2010-ongoing *P. pectinatus, C. globularis* 70%	Not yet reached	x	Kabus unpubl. data
12	Langer See		130	3.8/2.2	Since 1990: catchment restoration, improved wastewater treatment	?-1997-2001-?	?-2011-2015-ongoing *M. spicatum, C. demersum, N. marina*	not yet reached	x	Rücker et al., 2015, unpubl. data
13	Nonnensee		76	2.2/?	Re-flooded area (mid 1990's)	?- 2012 very few *P. pusillus*	2012, 2016 *P. pectinatus, M. spicatum, Ceratophyllum* spp.	not yet reached		Kabus unpubl. data
14	De Wittsee		24	2.1/1.4	Improvement of wastewater treatment	1970-2009 *Nuphar lutea*	2009-ongoing *E. nuttallii, Lemna*	not yet reached	x	Van de Weyer, unpubl. data
15	Steinhuder Meer		3000	2.9/1.35	Improvement of wastewater treatment	1960-1998 none	1999-2001: 2002-03: *E. nuttallii 2003-08: P. perfoliatus, P. crispus* 2009-?: shift back to turbid	not yet reached		Hussner et al., 2014
16	Felbrigg Lake	UK	2.7	1.3/0.9	Creation of pre-lake wetland in 2012 resulting in N-limited conditions. Cormorant predation on rudd	1960-2013 *P. pectinatus, P. pusillus, P. crispus, Z. palustris*	2014-ongoing *C. demersum, Chara* spp. *P. pectinatus, P. crispus, P. pusillus*	unclear whether already reached		Sayer et al., 2010a,b; Sayer et al. unpubl. data
17	Barton Broad		75	2/1	Progressive increase in number of effluents with P removal	1974-1990 none	1990-2000 *C. demersum, P. crispus* <1%	reached after sediment removal 1996 (Table 2, no. 41)	x	Phillips et al., 2005, 2015
18	Wolderwijd	NL	2650	2.5/1.5	1982-89: Flushing with nutrient-poor water	1969-1975 *P. pectinatus, P. perfoliatus* (Figure 5)	1976-1995 *P. pectinatus, P. perfoliatus, P. pusillus* (Figure 5)	reached after biomanipulation carried out since 1990 (Table 2, no. 25, Figure 5)	x	Scheffer et al., 1992; Noordhuis et al., 2016
19	Veluwe-meer		3400	5/1.55	1982-89: Flushing with nutrient-poor water	1975-76 *P. pectinatus* 0-5% (Figure 4)	1977-1995 *P. pectinatus, P. perfoliatus, P. pusillus, Characeae* 10-15% (Figure 4)	1996-now *P. pectinatus, C. aspera, C. contraria, N. obtusa* 30-85% (Figure 4)	x	Scheffer et al., 1992; Van den Berg et al., 1998, 1999; Noordhuis et al., 2016
20	Eemmeer		1520	?/2.1	Since 1995: improved sewage treatment and closure of treatment plant	1970-1999 *P. pectinatus* 1–5% (Figure 4)	2000-ongoing *P. crispus, P. pectinatus, P. pusillus* 10-40% (Figure 4)	not yet reached, but first Characeae visible since 2010 (Figure 4)	x	Noordhuis et al., 2016
21	Arresø	DK	3987	5.6/3.1	Improved sewage treatment, artificial lakes on the main inlet stream, reduced catchment.fertilization	1989-1996 none	?- 2011: *C. globularis, C. vulgaris, M. spicatum, P. perfoliatus, P. berchtoldii, P. cripus, P. pectinatus*	unclear whether already reached	x	Jeppesen et al., 2007a,b Søndergaard, unpubl. data

**Table 2:** The response of north temperate shallow lakes in The Netherlands (NL), Sweden (SE), Denmark (DK), United Kingdom (UK) ,and Germany (DE) to biomanipulation, natural fish kills or other lake-internal measures (x: data on Secchi disk transparency and total phosphorus concentrations were available and used in Figure 2B).

**No**.	**Lake**	**Country**	**Size (ha)**	**Depth (max/mean) (m)**	**Measures applied**	**Period of turbid conditions and major remaining macrophyte species**	**Periods of clear conditions, macrophyte species and coverage (% lake area)**	**Period of return to turbid conditions**	**Data Figure 2B**	**References**
22	Duiniger-meer	NL	30	?/1	1992,1993,1994: Fish removal	?-1992	1992-ongoing *Chara globularis, C. vulgaris Nitellopsis obtusa* (2000-04)	Varying macrophyte cover, but no shift back to turbid	x	Van Berkum et al., 1995; Meijer et al., 1999; Riegman, 2007; Verhofstad et al., 2017; Van Donk unpubl. data
23	Ijzeren Man		11	2.3/2.3	1989: Complete removal of fish biomass by pumping dry, restocking with pike fingerlings, roach, rudd, ide and tench, sediments removed	1960s−1989	Within 2 months of fish removal, macrophytes covered 50% of lake, *Characeae*	1995-?, varying macrophyte cover	x	Meijer et al., 1999; Van Donk unpubl. data
24	Noorddiep		4.5	?/1.5	1988: Biomanipulation	?-1989	1989-? *E. canadensis, C. demersum*	clear for at least 8 years despite TP 250 μg L^−1^	x	Meijer et al., 1999; Van Donk unpubl. Data
25	Wolderwijd		2650	5/1.5	1990: Biomanipulation	See Table 1 (lake no. 18)	1992- *C. contraria, C. vulgaris* Figure 5	none	x	Meijer and Hosper, 1997; Noordhuis et al., 2016
26	Zwemlust		1.5	2.5/1.5	1987: Lake drained empty, fish completely removed, restocked with pike and rudd 1999: Temporary lowering of water level, fish removal	?-1987	1988–1996 1988–1989: *E. nuttallii*, 1990–1991: *C. demersum* 1992–1994: *P. berchtoldii* 1995–1996: *E. Nuttallii*	1997–1999	x	Van de Bund and Van Donk, 2002; Verhofstad et al., 2017
27	Terra Nova		85	?/1.4	2003: (Removal of planktivorous and benthivorous fish)	1987–2003 1994: sparse stands of *C. demersum, P. lucens, P. obtusifolius, P. pectinatus, M. spicatum*	2004-? *C. demersum, E. nuttallii, P. obtusifolius, Nitella mucronata, N. marina, Utricularia vulgaris*			Van de Haterd and Ter Heerdt, 2007
28	Galgje		3.1	?/1.1	1987: Removal of all planktivorous and 85% of benthivorous fish in 1987	?-1988	1988: Within 2 months of fish removal macrophyte covered lake, *Characeae* 2014: *C. demersum, L. minor, P. crispus, S. polyrhiza*			Meijer et al., 1999; Verhofstad et al., 2017
29	Loender-veense Plas		270	?/2.7	2004/05: Removal of 95% of fish stock	1980s−2004 *P. perfoliatus*	2005-present *E. nuttallii, N. marina, C. globularis, C. connivens*	none		Pot and Ter Heerdt, 2014; Verhofstad et al., 2017
30	Naarder-meer		1,042	?/1.0	1993–1996: Sediment dredging	1980–1989 *Z. palustris, P. pectinatus*	1990–1995-? *Najas marina, M. spicatum, C. globularis, R. circinatus, C. demersum, N. obtusa*			Bootsma et al., 1999
31	Finjasjön	SE	1,100	12/2.7	since 1970: nutrient load reduction, 1987: suction-dredging of sediments, 1992-2014. removal of cyprinids (*Abramis brama, Rutilus rutilus*)	?-1994 *M. spicatum, E. canadensis, P. perfoliatus*	1995-? *E. canadensis (disappeared 1997), M. spicatum P. perfoliatus*	?		Annadotter et al., 1999; Strand and Weisner, 2001; Lage et al., 2015
32	Ringsjön (Western Bay)		1,480	5.4/3.1	1992: Removal of about 50% of cyprinid fish	?-1992	*P. crispus, P. perfoliatus, P. pectinatus, M. spicatum, P. lucens, E. canadensis*	2000–2005		Strand, 1999; Hansson unpubl. data
33	Vasatorp-dammen		2.1	1.4/1.1	1992: Fish removal by rotenone	1989–1992 none	1993–1996 *P. natans, P. obtusifolius, C. demersum, C. globularis*	?		Blindow et al., 2000
34	Væng	DK	15	1.9/1.2	1986-88 and 2007-09: Fish removal	?-1986	1989–1996 and 2010-now *E. canadensis*	1997–2009	x	Jeppesen et al., 1991; Søndergaard et al., 2017
35	Arreskov		317	3.7/1.9	1991: Fish removal	?-1991	1992–1998 *Characeae, P. pectinatus, P. pusillus, P. crispus, Z. palustris,C. demersum, Z. pedunculata*		x	Lauridsen et al., 2003b; Søndergaard et al., unpubl. data
36	Alderfen	UK	5.2	1.2/1	1979: Isolation from inflow, 1990: natural fish kill, 1992–1993: sediment removal, 1995, 2000: fish removal	Several turbid periods	*C. demersum* 20–65%	several phases of macrophyte decline (e.g., 1994, 1999–2000)	x	Moss et al., 1986, 1990; Perrow et al., 1997; Hoare et al., 2008; Phillips et al., 2015
37	Cockshoot Broad		5.5	1.2/1	1992: Isolation from river, sediment removal; 1989/90, 1996-2002, 2004-08: fish removal	1970–1980 none	1990-2012 *C. demersum, N. marina, Z. palustris* 40-58%	none	x	Moss et al., 1986; Hoare et al., 2008; Phillips et al., 2015
38	Hoveton Little Broad Pound End		15.5	1.5/1.0	1990: Suction dredging, 1990-1999: several fish removals from isolated bay (Pound End)	1970s−1991	1995-2006 *C. demersum, N. marina*	Low macrophyte abundance since 2001	x	Hoare et al., 2008; Phillips et al., 2015
39	Ormesby Great Broad		40	1.5/0.9	1995: Fish removal	1970–1989 *Chara, Z. palustris, P. pectinatus, C. demersum, M. spicatum, P. pusillus, P. crispus*	1995–2010 *C demersum, E. canadensis, Z. palustris, P. pectinatus, Chara, P. friesii* 67%	none	x	Phillips et al., 2015
40	Cromes		4.3	1.2/1	1988: Sediment removal, 1992: Barrier to isolate from river,1999: natural fish kill, 2004: Sediment removal	?	1995-ongoing *C. demersum* 80%		x	Perrow et al., 1997; Phillips et al., 2015
41	Barton Broad		75	2/1	1980: P reduction from effluents upstream 1996: sediment removal	see Table 1 (lake no. 17)	2000-2012 *C. demersum, P. pectinatus, E. canadensis* 12%		x	Phillips et al., 2005, 2015
42	Schollener See	DE	95	1/ < 1	2002: Natural fish kill during summer flood	~1980 to 2003 none	2004 *C. demersum, N. marina, P. berchtoldii, P. crispus*	2005-ongoing?	x	Knösche, 2008
43	Rangsdorfer See		272	2.5/1.5	2009/10: Natural winter fish kill	?-2009 (probably several decades)	2010–2011 *C. demersum, M. spicatum, P. crispus*	2012- ongoing		Hussner et al., 2014
44	Schwandter See		16.5	2.5/1.6	2002: P-precipitation with aluminum sulfate 2009/10: Natural winter fish kill	?-1995-2002 sparse stands of *C. demersum, P. crispus*	2003-2010 *C. demersum, P. crsipus* 100%	2011–2015-?: shift to turbid state after carp stocking	x	Mathes, 2007; Nixdorf et al., 2013; Hussner et al., 2014
5	Ivenacker See		73.3	1.9/1.1	2009/10: Winter fish kill 2012/13: sediment dredging	?-2009	2010-? *Characeae*	?	x	Nixdorf et al., 2013
46	Schloßsee Buggen-hagen		9.8	2.9/0.8	1990-96: Improved wastewater treatment, 1997: Sediment dredging	?-1997	1998-?, 2006-? *C. demersum, C. submersum. C. hispida*	2003	x	Mathes, 2007
47	Möllener See		18.7	2.2/2	2006: P-precipitation with aluminum sulfate	?	2007–2009-? *Characeae* (occurred 3 years after treatment)	?		Hussner et al., 2014
48	Bachtel-weiher		4.8	2/1.6	2002: Lake drained empty, fish completely removed, restocked with pike	?-2002	2003–2006-? *M. spicatum, R. trichophyllus, C. globularis, N. mucronata* 50-90%	?		Hussner et al., 2014
49	Herren-wieser Weiher		6.7	4.7/1.8	2001: Lake drained empty, partial sediment removal, fish completely removed, restocked with pikeperch	?-2001	2002 Mainly *R. circinatus, P. berchtoldii, P. crispus, P. lucens, P. pectinatus, C. contraria, C. globularis* 75%	2003–2005-? (after illegal carp stocking)		Hussner et al., 2014

**Figure 2:**
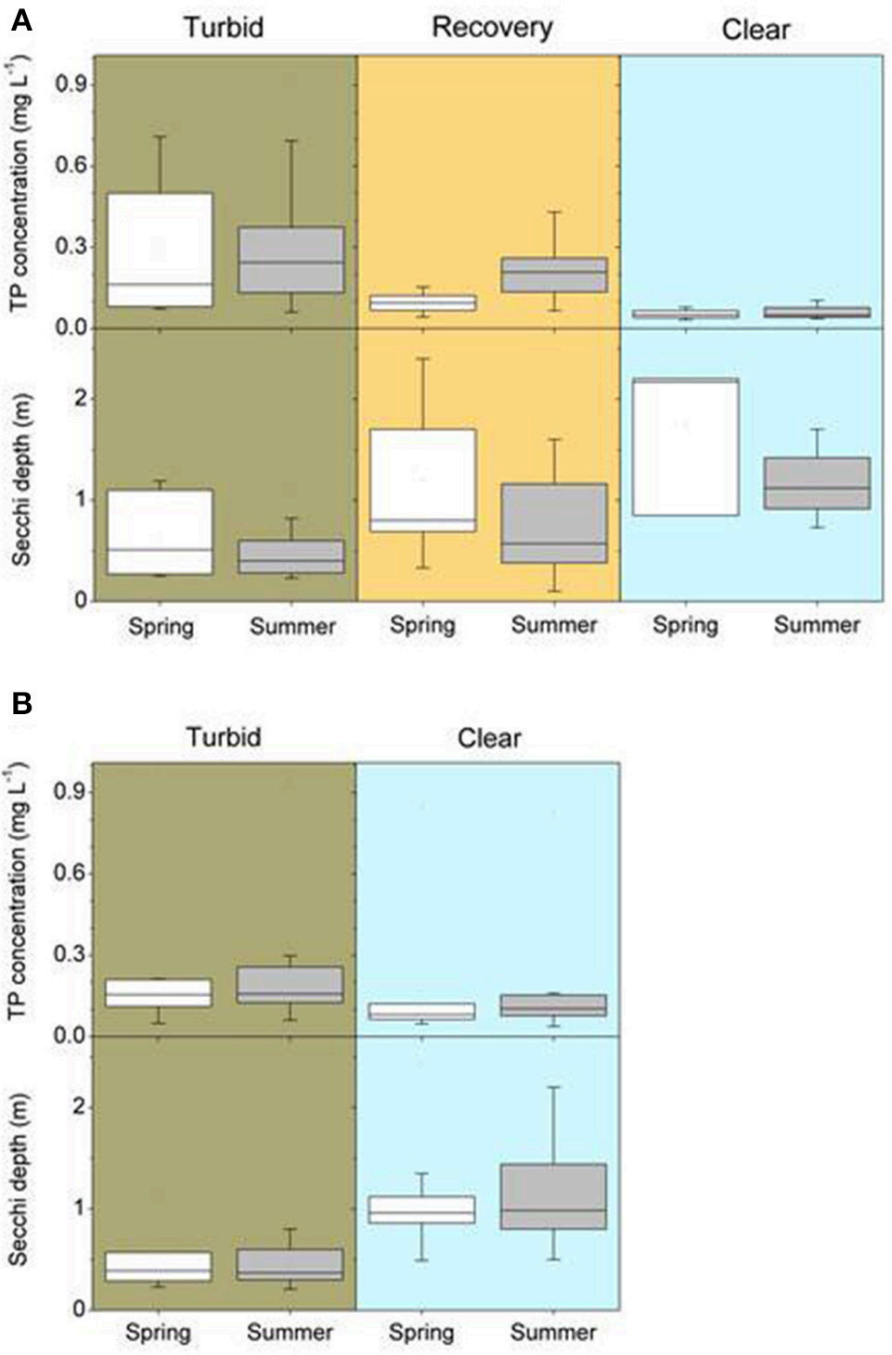
Total phosphorus (TP) concentrations and Secchi depth in spring (April–June) and summer (July–September) of different north temperate shallow lakes **(A)** before and after external nutrient load reductions during the turbid, the intermediate recovery and the clear-water state (for details see Table 1) and **(B)** before (turbid) and after (clear) biomanipulation or other lake-internal measures (for details see Table 2).

For all lakes, we retrieved information from the turbid period and its macrophyte assemblage, macrophyte composition after external/internal restoration, total phosphorus (TP) concentrations in the water and Secchi depth in spring (April-June) and summer (July–September) using published studies or questionnaire responses provided by co-authors. As is commonly the case, data on TP concentrations and Secchi depth were very diverse, ranging from multi-year weekly measurements to single values. The data were merged into a single value for each season and lake by using means (Figures 2A,B) and raw data are available as a supplement. We also analyzed the occurrence of dominant macrophyte species during the recovery period. To visualize potentially typical recovery patterns, the long-term changes in nutrient concentrations, water transparency and macrophyte occurrence are shown in more detail for three lakes restored only by reductions in external nutrient loading (Müggelsee, Veluwemeer, Eemmeer, for details see no. 1, 19 and 20 in Table 1) and for three lakes restored through biomanipulation of the fish community (Noorddiep, Wolderwijd, Zwemlust, for details see no. 24–26 in Table 2).

Mann-Whitney *U*-tests were performed to compare lake size, maximum and mean depths between lakes with different restoration measures (external nutrient load reduction vs. internal measures). Total phosphorus concentrations and Secchi disk transparency were compared between the different states (turbid, intermediate, clear) in lakes with external nutrient load reduction) using Kruskal-Wallis tests and subsequent posthoc comparisons (separately for different seasons). The same was done for lakes with internal measures using Mann-Whitney U tests. All statistical tests were run in SPSS.

### PCLake simulations

Simulating the response of water clarity and macrophyte biomass to external nutrient load reduction and detecting thresholds of nutrient loading for either intermediate recovery or clear state required adaptation of the established ecosystem model PCLake. This model has previously been used to estimate threshold responses of shallow lakes to nutrient loading (Janse et al., 2008; Janssen et al., 2017), and to simulate the response of temperate shallow lakes to climate warming (Mooij et al., 2007), to mowing of macrophytes (Kuiper et al., 2017) and—in a variant of the model with three plant species—to biomanipulation and herbivory (Janse et al., 1998). PCLake consists of a number of coupled ordinary differential equations that describe the most important biotic (submerged macrophytes, phytoplankton, detritivorous macrozoobenthos, zooplankton, zooplanktivorous fish, benthivorous fish, and piscivorous fish) and abiotic (detritus, inorganic material, dissolved phosphorus, ammonium, and nitrate) components of both the water column and the top-layer of the sediment in a non-stratifying shallow lake (Janse, 1997, 2005). All organic components (apart from predatory fish) are modeled in terms of dry weight (DW), nitrogen (N), and phosphorus (P), and hence the nutrient-to-dry-weight ratios of the organic components are variable. Internal fluxes of nutrients between the sediment layer and the pelagic zone, including internal loading, are accounted for and modeled dynamically.

For our simulations we used the default settings of a lake in PCLake. This default lake represents a relatively shallow lake with an average depth of 2 m and is relatively small with a maximum fetch of 1,000 m, an areal hydraulic loading of 20 mm days^−1^ (= 7.2 m year^−1^), no infiltration or seepage, no surrounding wetland zone, and a lightly clayish sediment (30% dry matter, of which 10% organic matter, and 10% fine mineral material) (Janse 2005). Due to small size and shallowness, the lake is dominated by macrophytes when nutrient loads are sufficiently low, but as nutrient loads increase the lake switches to a turbid state. This switch occurs rather suddenly due to the positive feedbacks in the model (Janse 2005) that lead to a critical transition (e.g., Scheffer and Carpenter, 2003). A common method to determine critical transitions is bifurcation analysis. In this approach the model is run to equilibrium several times, each with a different nutrient load. For each run, the yearly average phytoplankton chlorophyll-a concentration and macrophyte biomass are calculated. To assess the presence of hysteresis this procedure is repeated twice for each level of nutrient load, the first starting from a clear lake and the second from a turbid lake. Where the equilibrium outcomes of these two runs with identical nutrient load differ, hysteresis is inferred. Here we ran the model for nutrient loads ranging from 0.1 to 2.5 mg m^−2^ days^−1^ (0.4–9.0 kg ha^−1^ year^−1^) to cover a wide range of the eutrophication axis. The output of the bifurcation analysis is a load-response curve (or bifurcation plot) showing the effect of nutrient load on the biomass of primary producers. The point of a sudden switch marks the critical transition(s).

To simulate the influence of temperature and light on the response of different macrophyte species to nutrient load reduction we had to make two adjustments to the original formulations of PCLake, while maintaining the modeling of macrophytes as one functional group. First, the original power function for temperature limitation of macrophytes was replaced by a temperature optimum curve L_T_ (-):

$$L_{T}=e^{-\frac{0.5}{\sigma^{2}}\left[{\left(T-T_{opt}\right)}^{2}-(T_{ref}-T_{opt})\right]}$$

Here, σ (°C) is the temperature constant based on a Gaussian curve, *T* (°C) is the water temperature, *T*_*opt*_ (°C) is the optimum temperature for macrophytes and *T*_*ref*_ (°C) is the reference temperature used to normalize the limitation function to 1 (Janse, 2005). With this function the model is flexible to simulate macrophytes with different temperature optima. The second adjustment is the timing of root allocation which occurs in the autumn when macrophytes store energy to overwinter, for instance, in propagules. In the original PCLake, this timing was linked to a specific day in the year while submerged plants are known to respond to physiological and environmental cues, such as light availability, to determine timing of root allocation (Madsen, 1991). Hence, we decided to link the timing of root allocation to a minimum daily light availability for macrophytes, following Madsen (1991), Van Dijk and Van Vierssen (1991), and Van Dijk and Janse (1993). The available light for macrophytes is based on the light availability over the depth of the water column corrected for periphyton shading which is estimated by:

$$\bar{PAR}=PAR_{0}\;*D-\frac{LN\left(\frac{PAR_{0}}{PAR_{bot}}\right)}{D}*\left(1-\varepsilon_{periphyton}\right)$$

In which *PAR* (W m^−2^) is an approximation of the average Photosynthetic Available Radiation (PAR) for plant photosynthesis, *PAR*_0_ (W m^−2^) is the PAR available at the top of the macrophyte layer, *PAR*_*bot*_ (W m^−2^) is the PAR available at the bottom of the macrophyte layer, D (m) is the depth and ε_*periphyton*_ is the shading by periphyton. In order to restrict complexity, we refrained from adding periphyton as an extra compartment to the model, but instead used the empirical relationship of Vadeboncoeur et al. (2006) to estimate periphyton chlorophyll-a biomass:

$$LOG_{10}\left(Chla_{periphyton}\right)=c_{1}*{LOG}_{10}\left(TP\right)+c_{2}$$

where *c*_1_ = 1.79 and *c*_2_ = 0.85 and TP is the in-lake total phosphorus concentration (mg m^−3^). The periphyton chlorophyll-a biomass is then used to estimate the shading effect:

$$\varepsilon_{periphyton}=Chla_{periphyton}*S*f\left(L\right)*f\left(T\right)*f\left(\alpha \right)$$

where *S* is the specific light attenuation by periphyton (m g^−1^) and correction factors for light limitation *f(L)*, temperature limitation *f(T)* and available plant surface area for periphyton growth *f(*α*)*. The shading effect of periphyton affects the timing of root allocation through light availability to macrophytes as well as the light limitation of macrophyte shoots.

PCLake has previously been calibrated following a Bayesian approach to parameter estimation and uncertainty analysis using data from nearly 40 temperate shallow lakes (Janse et al., 2010). Although this calibration did not account for the specific effect of periphyton, the data used for model calibration most likely integrate this effect indirectly (Kuiper, 2016). By adding the effect of periphyton to PCLake implicitly, we thus have to technically recalibrate the model. This implies adjusting the parameter settings of the model, such that given the same boundary conditions, the model produces the same output. Therefore we have calibrated the adjusted model manually by lowering the half saturation light constant of vegetation and decreasing the parameter for dark respiration of vegetation (see Table 3 for new and calibrated parameter settings). PCLake is implemented in DATM (Mooij et al., 2014). For the full overview of parameter settings and model formulations, please see the DATM-file in the Supplementary Material.

**Table 3:** Parameter settings for PCLake.

**Parameter**	**Description**	**Unit**	**Value^a^**	**Source**
σ	Temperature constant based on a Gaussian curve	°C	20	-
T_opt_	Optimum temperature for macrophytes	°C	20	-
T_ref_	Reference temperature	°C	20	–
c_1_	Slope of logistic curve periphyton	–	1.79	Vadeboncoeur et al., 2006
c_2_	Intercept logistic curve periphyton	–	−0.85	Vadeboncoeur et al., 2006
S	Light attenuation by periphyton	m g^−1^	0.03	Van Dijk, 1993
L_min_	Minimal light availability cue needed for plants to initiate increased root allocation	W m^−2^	91.2	Calibrated
h_Lveg_	Half saturation light constant of vegetation at 20°C	W m^−2^	12 (17)	Calibrated
kD_resp_	Dark respiration rate of vegetation	day^−1^	0.015 (0.02)	Calibrated

a *Values between brackets are the original value*.

## Results

### Lake water quality following external and internal restoration

Our literature review provided information on water quality and macrophyte development in 21 turbid lakes that were subject to external nutrient loading reduction without additional in-lake measures (Table 1) and 28 lakes with in-lake restorative measures. Some of these measures were preceded or accompanied by external nutrient loading reduction (Table 2).

Lakes with internal measures were on average smaller than lakes with external nutrient load reduction alone, while maximum depths were higher but mean depths were similar (Table 4). Turbid conditions lasted from 1 year (Lake Veluwe) to 51 years (Dümmer). Often, however, exact timing and duration of the turbid period are unknown (Tables 1, 2). During the turbid phase, spring and summer TP concentrations were high (~ >0.15 mg L^−1^), while Secchi disk transparencies were low (~0.4 m), with considerable differences between lakes (Figures 2A,B, Table 4).

**Table 4:** Size, maximum and mean depths, total phosphorus concentrations and Secchi disk transparency in lakes after external nutrient load reduction or implementation of internal measures.

**Lakes**	**State**	**Size (ha)**		**Maximum depth (m)**		**Mean depth (m)**		**Total phosphorus concentrations (μg L**^**−1**^**)**				**Secchi disk transparency (m)**			
								**spring**		**summer**		**spring**		**summer**	
		**mean**	**median**	**mean**	**median**	**mean**	**median**	**mean**	**median**	**mean**	**median**	**mean**	**median**	**mean**	**median**
External nutrient load reduction	Turbid	984	450	5.0	3.9	2.4	1.8	281	163 A	317	244 A	0.66	0.45 A	0.46	0.4 A
	Intermediate							97	94 A	206	206 A	1.23	1.11 AB	0.75	0.6 AB
	Clear							53	51 B	59	49 B	1.74	2.17 B	1.17	1.12 B
*P* (Kruskal-Wallis test)									0.02		0.007		0.042		0.02
Internal measures	Turbid	273	18	2.8	2.1	1.5	1.3	229	156 a	256	150 a	0.62	0.45 a	0.45	0.40 a
	Clear							168	81 a	170	103 b	1.12	0.96 b	1.10	0.99 b
*P* (Mann-Whitney U test)		<0.001		0.016		0.076			0.051		0.011		0.026		<0.001

*Size and depths include all lakes, while TP and transparency data were only available for selected lakes (see Figure 2, Tables 1, 2). Different letters indicate significant differences between values of different states (external nutrient load reduction: Kruskal-Wallis test, capital letters; internal measures: Mann-Whitney U-test, small letters)*.

**Figure 3:**
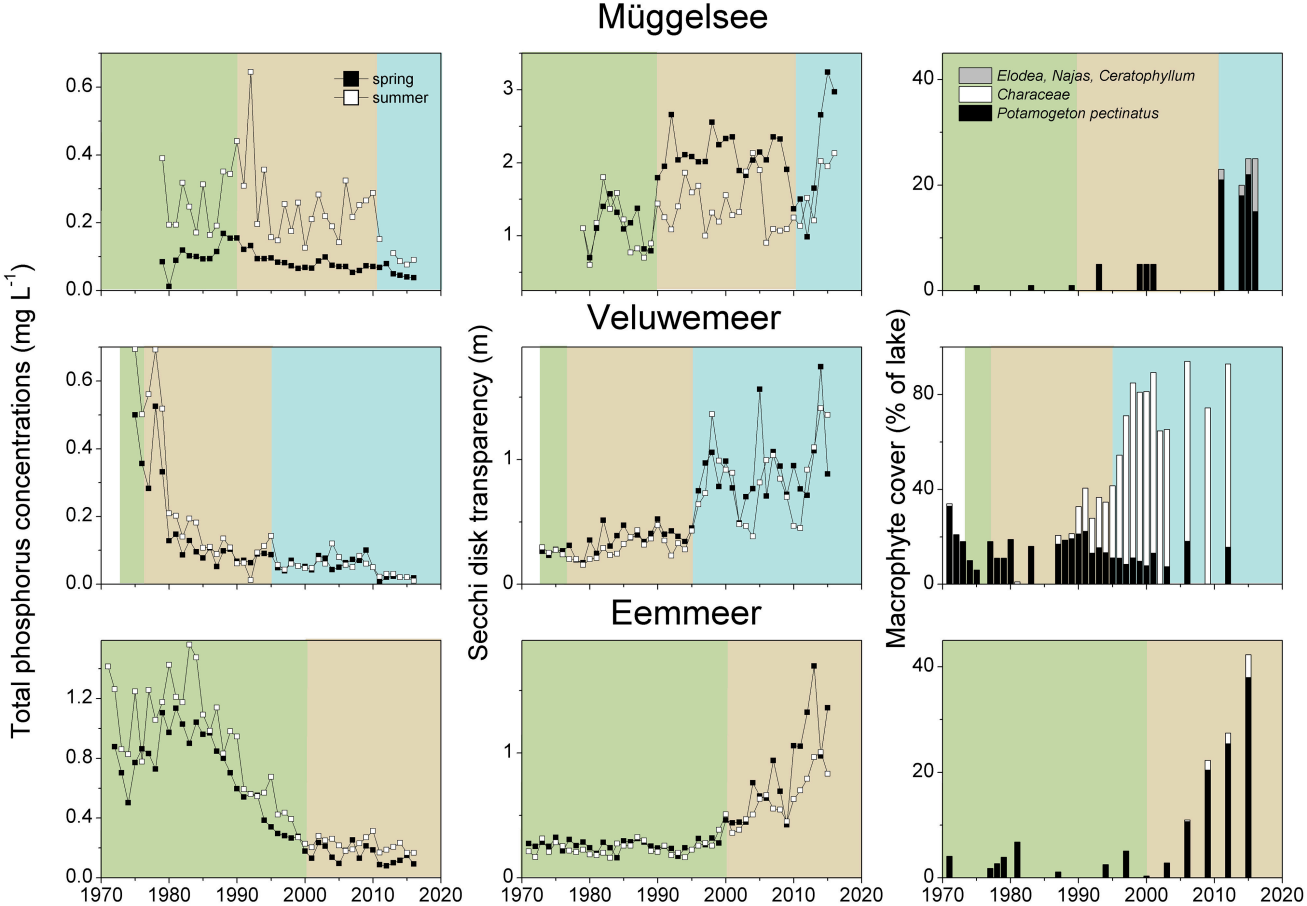
Total phosphorus (TP) concentrations and Secchi depth in spring (April–June) and summer (July–September) and macrophyte coverage in Lake Müggelsee; Lake Veluwe and Lake Eem during the turbid (green), the intermediate recovery (brown) and the clear-water (blue) state (for lake details see Table 1).

External nutrient loads were usually reduced following improved sewage treatment in the catchment. Lake Veluwe and Wolderwijd were flushed with nutrient-poor water (Table 1). The most commonly applied internal measure was biomanipulation in the form of removal of benthivorous and planktivorous fish (in some cases by draining/pumping the lake dry). In nine lakes, sediment removal was applied as an additional or the sole measure and in five lakes natural fish kills occurred during severe winters or as a result of a summer flood (Table 2), emulating the effects of planned biomanipulation. During the first years after nutrient load reduction, TP concentrations were lower than during the turbid period, but still about twice as high in summer as in spring. Spring water transparency was higher than during the turbid period, while summer values were still low (Table 4, Figure 2A). This phase has been found to last up to 20 years (e.g., Müggelsee), but often its start has not been recorded and/or lakes have not yet reached stable clear conditions (Table 1). Lakes Müggelsee, Veluwemeer, and Eemmeer show a similar intermediate recovery state with spring water transparencies being higher than during turbid summer conditions which lasted for about 20 years (Figure 3). A switch back from intermediate to turbid conditions has only been observed in Lake Steinhuder Meer, in this case about 10 years after macrophytes returned (Table 1).

**Figure 4:**
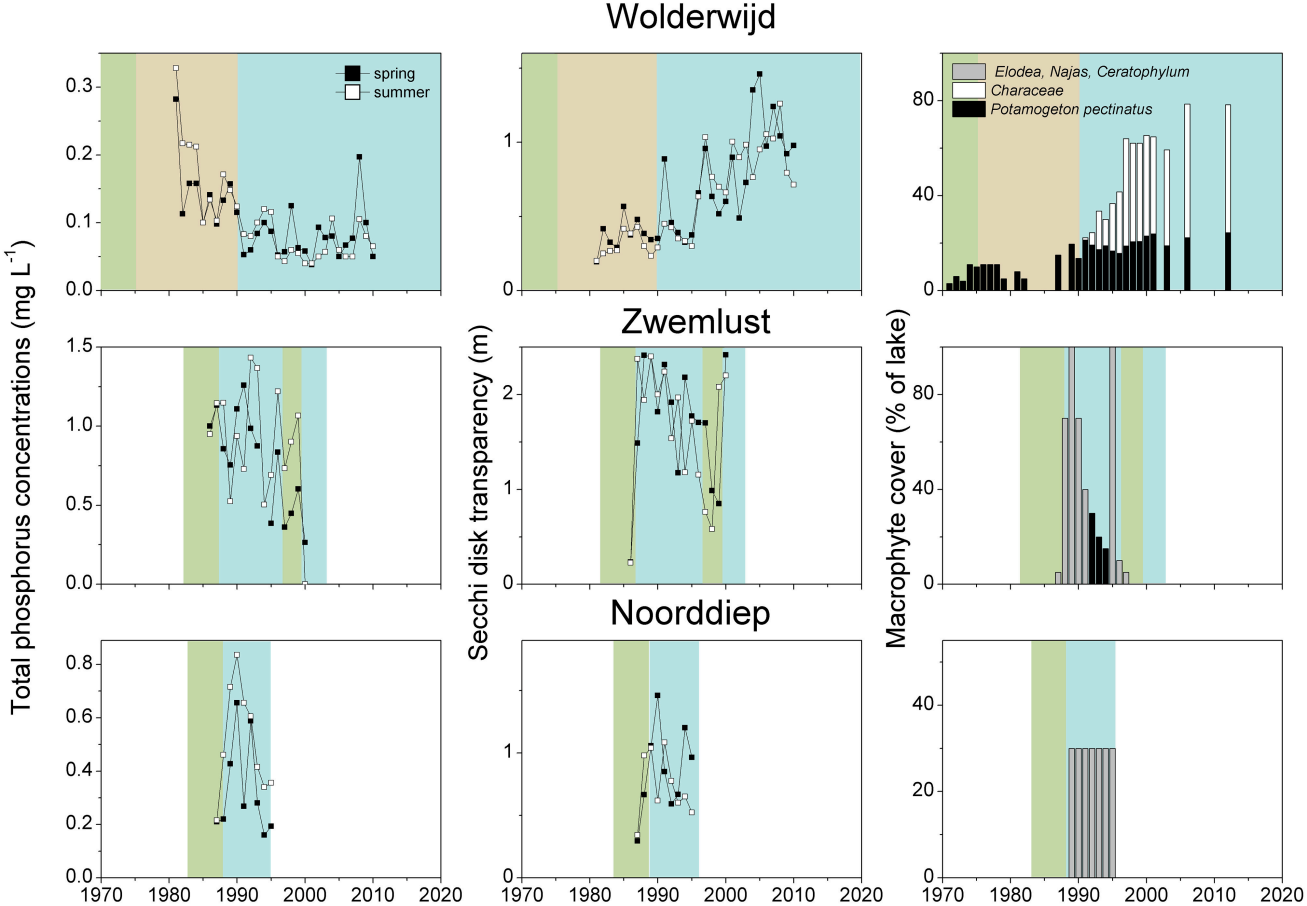
Total phosphorus (TP) concentrations and Secchi depth in spring (April–June) and summer (July–September) and macrophyte coverage in Lake Wolderwijd, Lake Zwemlust and Lake Noorddiep before (green and brown) and after (blue) biomanipulation (for lake details see Table 2). In Lake Zwemlust, *P. berchtoldii* occurred instead of *P. pectinatus*, and the coverage in Lake Noorddiep was only estimated based on the information that it was higher than 25% (Gulati and Van Donk, 2002).

After implementation of in-lake measures, TP concentrations were at the same level as before, in both spring and summer, whereas water transparency in spring and summer was significantly higher than before restoration (Table 4, Figure 2B). These unstable clear conditions often only lasted for a few years and many lakes shifted back to turbid conditions (e.g., no. 23, 26, 32, 34, 36, 38, 42, 43, 44, 46, 49 in Table 2). Fish stock reductions in Wolderwijd, Zwemlust and Noorddiep led to clear conditions both in spring and summer (Figure 4). Lake Zwemlust shifted back to turbid conditions after 9 years, while Noorddiep stayed clear for at least 8 years with no further information on subsequent periods.

Only six of the lakes with external nutrient load reduction (~25%, no. 1, 3, 7, 10, 18, and 19 in Table 1) reached stable clear-water conditions with lower ambient TP concentrations and higher Secchi depths than during the intermediate recovery state both in spring and summer (Figure 2A, Table 4). In four of the 28 lakes (14%, no. 25, 29, 37, 39 in Table 2, Lake Wolderwijd in Figure 4), stable, longer-term clear-water conditions with a diverse macrophyte flora were obtained after the application of in-lake measures. For several lakes, their longer-term development is not known (Table 2).

### Model simulations on lake response to external nutrient load reduction

**Figure 5:**
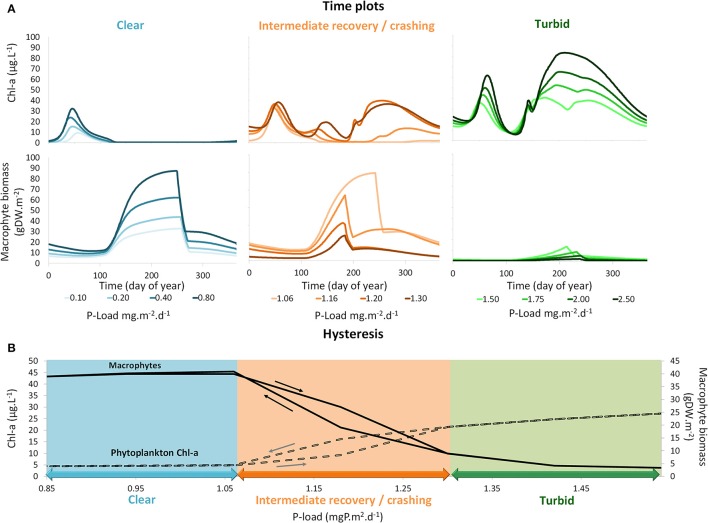
**(A)** Time series of simulation using PCLake of chlorophyll-*a* concentrations and macrophyte shoot biomass for different phosphorus (P) loadings within the clear, intermediate recovery and turbid states. Darker colors are associated with higher P loading simulations. **(B)** Hysteresis plots showing yearly mean simulated values of chlorophyll-*a* concentrations and macrophyte shoot biomass for different phosphorus (P) loadings within the clear, intermediate recovery and turbid states. Arrows denote the directions of the hysteresis effects.

The results of the simulations using the adjusted PCLake model revealed three stages for lakes that undergo external nutrient load reduction: a turbid state, an intermediate recovery state and a clear state (Figure 5A). For the default lake in PCLake, the turbid state occurs if the P load exceeds 1.3 mg P m^−2^ days^−1^. The intermediate recovery state occurs between the two critical transitions that appear at a P load of 1.06 and 1.3 mg P m^−2^ days^−1^. If the P load drops below 1.06 mg P m^−2^ days^−1^ the lake turns into a clear state. The critical P loads for the intermediate recovery state are smaller in the adjusted model including periphyton than with the original formulation of PCLake (critical transition between approximately 1 and 2 mg P m^−2^ days^−1^, Janse et al., 2008). The periphyton effect thus reduces the threshold for intermediate recovery state of the default lake by about a quarter of what it would be without periphyton. The reason for this is that in case of hysteresis, the position of the highest critical nutrient load is mainly determined by macrophyte characteristics, while the position of the lowest critical nutrient load is mainly determined by phytoplankton characteristics. This is in agreement with the results of the sensitivity analysis of PCLake (Janse et al., 2010). Since periphyton negatively affects the performance of macrophytes, the highest critical nutrient load was reduced, while phytoplankton characteristics were unaltered and the lowest critical nutrient load (1 mg P m^−2^ days^−1^) thus did not change.

The turbid state was characterized by lack of macrophytes and enhanced phytoplankton biomass with increasing nutrient loading (Figure 5A). The intermediate state was characterized by a changed phenology of the primary producers. With increasing nutrient loading, the phytoplankton summer peak shifted to earlier dates of the year and this advancement of phytoplankton was mirrored by an abbreviated macrophyte growing season (Figure 5A). The shorter growing season was a direct effect of the inclusion of periphyton shading in our adapted version of the model. The shading of macrophytes by periphyton was most severe at the peak of summer when the light input and water temperature are high. As a result, macrophyte growth was limited to the period just after the clear water phase in spring until the start of the summer phytoplankton bloom. The clear state was characterized by an increase of macrophyte biomass with increasing nutrient loading (Figure 5A). Phytoplankton production was restricted to the spring bloom peak and there was no summer bloom.

The bifurcation plot (Figure 5B) shows a less sudden transition of macrophytes and phytoplankton compared to the abrupt transition between the phytoplankton-dominated turbid state and the macrophyte-dominated clear state, often seen in bifurcation plots in literature (e.g., Janse et al., 2008). The gradual course of the bifurcation plot is due to the inclusion of periphyton shading of macrophytes. This shading permits high biomass of both macrophytes and phytoplankton within the same year during the crashing or intermediate recovery phase. Furthermore, the region of hysteresis is tilted, leading to a less abrupt and thus more realistic critical transition from phytoplankton dominance to macrophyte dominance and back again.

### Macrophyte species recovery following external and internal restoration

During the turbid phase, three lakes with subsequent external nutrient load reductions were reported to lack macrophyte stands altogether and six and one lakes had sparse stands of *Potamogeton pectinatus* (also known as *Stuckenia pectinata*) and *P. pusillus*, respectively, while no information was available for the remaining lakes (Table 1). For lakes with internal measures, information on macrophyte species present during the turbid phase is available for 10 lakes. Apart from *P. pectinatus* and *P. perfoliatus*, plants such as *Ceratophyllum demersum, M. spicatum* or *E. canadensis* are mentioned, if indeed plant stands were present at all (Table 2).

**Figure 6:**
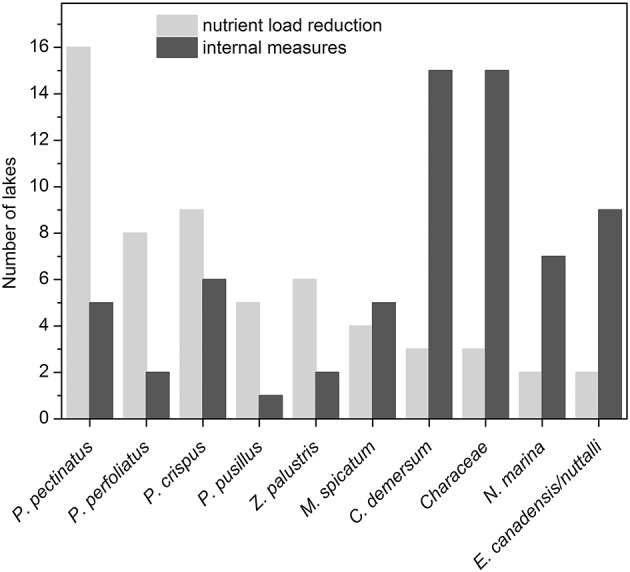
The 10 most common macrophyte taxa in north temperate shallow lakes during re-colonization after reduction of external nutrient loading (light gray) or implementation of internal measures (dark gray; for more details see Tables 1, 2).

During the intermediate recovery state *P. pectinatus* was the dominant macrophyte species in two thirds of the analyzed lakes with reduced external nutrient loading. Other pondweed species such as *P. perfoliatus, P. crispus* and *P. pusillus* or *Zannichellia palustris* were also found in several lakes during the intermediate recovery state, while other groups such as Characeae or *Elodea* species were much less common (Table 1, Figure 6). Lakes Müggelsee, Veluwemeer and Eemmeer were all dominated by *P. pectinatus* during the intermediate recovery state which lasted for about 20 years (Figure 3). Müggelsee and Veluwemeer seem to have entered a stable clear state with more diverse submerged vegetation (including Characeae in Veluwemeer) in 2011 and 1996, respectively, while Eemmeer has not yet reached that phase despite the recent detection of Characeae (Figure 3). Three other lakes also reached a stable clear state and were colonized by different species of Characeae and/or *Najas marina, Elodea* species and *C. demersum* (Table 1).

The dominant macrophytes occurring after lake internal measures were Characeae, *C. demersum, Elodea* species or *N. marina* (Table 2, Figure 6). Often, lakes had only one or two dominant species, and in at least 10 cases, lakes switched back to turbid conditions and lost their macrophytes again (Table 2, Figure 4). The response of macrophytes occurred gradually in Wolderwijd, showing an increase in Characeae coverage, while in the much smaller Zwemlust and Noorddiep, macrophytes immediately covered large areas, *E. nuttallii, E. canadensis* and *C. demersum* dominating (Figure 4, Table 2). In four lakes, stable clear-water conditions were obtained after in-lake measures, all being characterized by Characeae dominance (Table 2, Figure 4).

## Discussion

Our analysis of long-term data from 49 temperate shallow lakes during their recovery from a turbid phase reveals that both a reduction of the external nutrient loading and implementation of lake-internal measures often result in the occurrence of an intermediate state (Figure 1) that can last for several decades. External nutrient load reductions are often followed by the re-occurrence of a spring clear-water phase that opens a “window of opportunity” for macrophyte re-colonization, but with only a short growth period due to turbid conditions during summer. This pattern was confirmed by our model simulations (Figure 5). As hypothesized, macrophyte re-establishment following nutrient load reduction occurs in a reversed sequence to the one described for eutrophication by Sayer et al. (2010a,b). In contrast, lake internal measures such as fish or sediment removal often result in clear-water conditions during spring and summer. This clear-water state is, however, often only temporary and lakes frequently shift back to turbid conditions one or a few years after the restoration. Likely, the duration of the clear-water conditions is related to nutrient loading and the intensity of the restoration effort (Hansson et al., 1998). Only in a few examples have longer lasting clear-water conditions been observed. These required spring and summer TP concentrations below 0.05 mg L^−1^.

We also have evidence for our second hypothesis, namely that different types of restoration measures influence the macrophyte community composition. *P. pectinatus* and a few other pondweeds most often recolonise temperate shallow lakes with reduced external nutrient loading and dominate during the intermediate recovery state. The implementation of internal restoration measures results in the establishment of a different community, often consisting of a small selection from either hornwort (*C. demersum*), charophytes, water weeds (*E. canadensis, E. nuttallii*) or naiad (*N. marina*). Only in a few cases have lakes reached a state of clear-water conditions during spring and summer and with a more diverse macrophyte community.

### Submerged macrophyte survival during the turbid phase

Whether and which macrophyte stands or propagules survive during the turbid phase depends on the occurrence of macrophyte species before the shift to turbid conditions and on the length and severity of the turbid phase (e.g., Vari and Toth 2017). Seed banks in shallow lake sediments have often been assumed to be insufficient for recovery of submerged vegetation by germination, due to low numbers of viable seedlings, strong seed dormancy, strict germination cues and the reliance of many species upon vegetative reproduction (Haag, 1983; Kautsky, 1990; Rodrigo et al., 2013; Baldridge and Lodge, 2014). In contrast, De Winton et al. (2000) and Verhofstad et al. (2017) have shown that seed banks from even the most degraded lakes are capable of an emergence response and thus offer a potential means to restore vegetation. In our survey, the duration of complete macrophyte loss was often unrecorded, but periods of several decades are common (Tables 1, 2). If macrophyte stands survived during this period and were recorded, these were often sparse stands of *P. pectinatus*, a species commonly associated with the crashing phase during eutrophication (Sayer et al., 2010a). This species survives in very shallow water even under phytoplankton dominance (Hilt et al., 2013) and its strongly apical growth form may allow it to survive at greater depths in turbid water. The shallow littoral, especially in larger lakes, is strongly disturbed by wave action and only species with high anchorage and breaking strength can survive under these conditions (Schutten et al., 2005). *Potamogeton pectinatus* has a high breaking strength (Brewer and Parker, 1990) and its phenotypic plasticity allows it to form short plants in shallower water (Idestam-Almquist and Kautsky, 1995). In contrast, other common species in eutrophic lakes such as *E. canadensis* or *M. spicatum* have been described as deep water species with lower tensile strength (Brewer and Parker, 1990), while *C. demersum* has no roots for anchorage. These species are thus less likely to persist through severely turbid states in very shallow littoral areas and to serve as remnant populations for re-colonization, at least in larger lakes. Nevertheless, they have been reported during turbid phases in four lakes included in our survey, most probably in wind-protected areas or bays.

Knowledge of the survival of propagules in sediments during turbid phases is limited. In general, charophyte oospores and macrophyte seeds have been found to survive up to 150 years (Kaplan and Muer, 1990; De Winton et al., 2000; Alderton et al., 2017). Germination tests with sediments have been suggested before implementing lake restoration measures to forecast the potential for macrophyte recovery from internal sources (Hilt et al., 2006), however, these are not routinely applied. Re-colonizing macrophyte clones of several species that originated from periods before eutrophication have been found (Sand-Jensen et al., 2008), but knowledge about the origin of re-colonizing macrophytes post-restoration remains scarce (Bakker et al., 2013).

### Response of macrophytes to nutrient load reductions

Shallow temperate lakes often show a relatively rapid response to reductions in external phosphorus loading, characterized by a reduction in phytoplankton biomass during spring and early summer (Jeppesen et al., 2005). During late summer, however, the response is delayed because of sustained remobilisation of phosphorus from the sediment (Søndergaard et al., 2013). As a consequence, high phytoplankton and cyanobacterial abundance are often reasserted in summer (Sommer et al., 2012) resulting in turbid water and preventing macrophyte growth. In our survey, such conditions occurred at spring TP concentrations of around 0.1 mg L^−1^, while summer concentrations were twice as high.

The increased water transparency in spring and early summer seems to be exploited by certain macrophyte species, in our survey mainly *P. pectinatus* along with *P. perfoliatus, P. crispus* and *Z. palustris*. These macrophyte species are characterized by specific traits that may explain their prevalence. Firstly, they can compress their whole life cycle into the short clear-water period in spring and early summer due to early germination from tubers, turions or seeds, shortening time to peak biomass and allowing early formation of overwintering tubers and seeds (Table 5). Secondly, they can have short growth forms that can establish in very shallow habitats (e.g., Van Vierssen, 1982a). Thus, they are often the species that survive in turbid conditions in shallow margins (see Submerged Macrophyte Survival during the Turbid Phase) and then expand into deeper water with improvements in clarity during nutrient load reduction. Rhizomatic growth from remaining *P. pectinatus* stands has been shown, via microsatellite analyses, to be the dominant re-colonization mode in Lake Müggelsee; more recently established *P. pectinatus* stands had lower genotype diversity and were comprised of only a small subset of genotypes from shallower areas (Hilt et al., 2013). Thirdly, energy reserves in vegetative propagules such as tubers of *P. pectinatus* allow early onset of growth independent of light availability (Spencer, 1986). In addition, *P. pectinatus* can concentrate large parts of its biomass just under the water surface and thus survive in relatively turbid water (Van Wijk, 1988). An initial colonization of formerly turbid lakes with *P. pectinatus* during recovery has also been observed in deeper, stratifying lakes. Thus, in Lake Tegel (Germany), this species dominated for more than 20 years after the start of phosphorus stripping in the major inflow (Hilt et al., 2010).

**Table 5:** Characteristics of macrophyte species/groups typically recolonising temperate shallow lakes after external nutrient load reduction or following implementation of lake-internal measures such as biomanipulation.

**Restoration measure**	**Macrophyte species**	**Re-production mode**	**Germination**	**Colonization mode**	**Timing of peak biomass**	**Specific features**	**Epiphyton density**	**Allelopathic activity**	**Susceptibility to herbivory**
External nutrient load reduction	*Potamogeton pectinatus*	Mainly by tubers, although seeds are formed^1^	Between 10-15°C^7^	Rhizomatic growth^11^	June^19^	Concentration of biomass below water surface^1^ Tubers rich in carbohydrates^32^	Low^13^	Low^2^	High^3^^,^^14^^,^^28^
	*P. perfoliatus*	Turions^29^, seeds		Rhizomatic growth^11^					High^20^
	*P. crispus*	Mainly by turions^26^	Turions can sprout in late summer, but shoot elongation after winter above 10°C^26^	Turions	Late spring to early summer^25^			Low^2^	
	*P. pusillus*	Turions^4^		Rhizomatic growth^4^^,^^12^			Low^13^	Low^2^	High^14^
	*Zannichellia palustris*	Seeds^5^	Mainly temperature-dependent, above 12-16°C^5^^,^^27^	Seeds^12^		Short, but numerous shoots allow development in very shallow water^27^ Tolerant to disturbance by wave action^27^	Low^13^	Low^2^	
Internal measures	*Elodea canadensis*	Vegetatively^6^	May-June^22^, can be evergreen	Fragments, vegetative growth (peripheral propagation)^4^^,^^12^		Sudden biomass collapses^6^^,^^8^^,^^9^	High^13^	Medium^2^	Medium^3^
	*E. nuttallii*	Vegetatively^17^	Can be evergreen	Fragments, vegetative growth^12^	Aug.-Oct.^21^	Extremely high reproductive capacity^16^^,^^17^ Sudden biomass collapses	High^13^ Low^15^	Medium^2^	Medium^18^
	*Ceratophyllum demersum*	Vegetatively (dormant apices)	April^22^, can be evergreen	Fragments, vegetative growth^12^	August^31^	Sudden biomass collapses^10^	Low^15^	High^2^	Low
	*Najas marina*	Annual, seeds	Late in (temperate) season (>20°C)^30^	Seeds^11^	Mid August^11^			Medium^2^	Low^18^
	Characeae	Oospores	Can be evergreen, overwintering by shoot apices	Oospores	Late summer^23^		Low^15^	Medium^2^	Medium^3^

1 Van Wijk (1989),

2 Hilt and Gross (2008),

3 Dorenbosch and Bakker (2011),

4 Barrat-Segretain and Bornette (2000),

5 Bytnerowicz and Carruthers (2014),

6 Simberloff and Gibbons (2004),

7 Madsen and Adams (1988),

8 Rørslett et al. (1985),

9 Strand and Weisner (2001),

10 Van de Bund and Van Donk (2002),

11 Vari and Toth (2017),

12 Capers (2003),

13 Lalonde and Downing (1991),

14 Hidding et al. (2010),

15 Grutters et al. (2017),

16 Simpson (1990),

17 Josefsson (2011),

18 Pot and Ter Heerdt (2014),

19 Körner (2001),

20 Choi et al. (2002),

21 Best and Dassen (1987),

22 Best (1977),

23 Talling and Parker (2002),

24 Lillie et al. (1997),

25 Woolf and Madsen (2003),

26 Tobiessen and Snow (1984),

27 Van Vierssen (1982b),

28 Weisner et al. (1997),

29 Wolfer and Straile (2004),

30 Van Vierssen (1982c),

31 Best (1982),

32 *Spencer (1986)*.

Usually, the maximum colonization depth of macrophytes during the intermediate recovery phase is low (around 1 m) and consequently, depending on the lake morphometry, only small parts of the lake bed might be covered. In contrast, very shallow lakes may reach over 50% cover (Table 1). This suggests that in “deeper” shallow lakes, macrophyte coverage during the intermediate phase may be insufficient to stabilize clear-water conditions during later summer. Based on the findings of Søndergaard et al. (2016) submerged macrophyte coverage on average needs to pass a threshold of 20% of lake area to markedly lower phytoplankton densities. In principle, small stands can be sufficient as a refuge for phytoplankton-grazing zooplankton against fish predation (Lauridsen et al., 1996; Portielje and Van der Molen, 1999). However, abundant colonial and filamentous cyanobacteria which often dominate the summer phytoplankton communities during lake recovery cannot be effectively controlled by zooplankton grazers (Wang et al., 2010 and references therein). Bottom-up stabilizing mechanisms of macrophytes on water clarity such as nutrient competition, increased sedimentation within stands and reduced sediment resuspension will be inefficient at low plant coverage (Blindow et al., 2014). Low coverage is, however, not the only reason why macrophytes in the intermediate recovery phase cannot stabilize clear-water conditions in late summer, as shown in the case of the very shallow Lake Dümmer, where cyanobacteria blooms still occurred in summer despite high macrophyte coverage.

Our model simulations suggest that high periphyton shading triggers macrophyte disappearance in summer. Periphyton shading, often accompanied by herbivory (Hidding et al., 2016), has been shown to impair macrophyte development in empirical studies (e.g., Jones et al., 2002; Jones and Sayer, 2003; Roberts et al., 2003) and is argued to be a major factor in the failure of macrophytes to establish even decades after the start of nutrient loading reduction, despite suitable water clarity for plant re-establishment in spring (Phillips et al., 2005). In our adapted PCLake model, periphyton biomass was dependent on TP concentrations in the water, based on the positive correlation between chlorophyll content of periphyton on hard substrata and TP in the water column (Vadeboncoeur et al., 2006). In eutrophic shallow, temperate lakes, periphyton is often top-down controlled by a cascading effect from omnivorous fish that feed on periphyton grazers such as snails and chironomid larvae (Jones and Sayer, 2003). Thus, nutrient load reductions will only reduce periphyton shading after the fish biomass built up during the turbid period has also been reduced, which may take 10–15 years (Jeppesen et al., 2005). Furthermore, the observed dominant macrophyte species in the intermediate recovery phase after nutrient load reduction (Table 1) show little or no allelopathic activity that might hamper periphyton growth (Table 5), thus making them more susceptible to shading by periphyton.

Cyanobacteria have been shown to potentially inhibit submerged macrophyte growth via allelopathy (Zheng et al., 2013), but whether this mechanism contributes to the disappearance of macrophytes during the recovery phase in summer is unknown. Most of the dominant macrophyte species during intermediate recovery after nutrient load reductions are also highly susceptible to herbivory due to their low content of polyphenols, low carbon to nitrogen ratio and low dry matter content (Elger and Willby, 2003; Dorenbosch and Bakker, 2011, Table 5). Periphyton shading may further increase the sensitivity of macrophytes to herbivory (Hidding et al., 2016). Finally, fine-leaved species such as *P. pectinatus, P. pusillus* and *Z. palustris* also suffer from leaf plucking by omnivorous fish during periods of low zooplankton abundance when those fish switch to macroinvertebrate prey found in the periphyton of macrophytes (Körner and Dugdale, 2003). Such leaf plucking by fish can lead to a considerable leakage of nutrients from injured macrophyte tissue, thereby further stimulating phytoplankton growth (Hansson et al., 1987). Overall, while being well-suited for survival during turbid phases and for exploiting the clear-water conditions in spring for re-colonization, other traits of macrophyte species typical of the intermediate recovery phase following nutrient load reduction prevent their survival during later summer (Table 5).

Stable clear-water conditions in spring and summer with more diverse macrophyte vegetation were observed when both spring and summer TP concentrations reached about 0.05 mg L^−1^. This value corresponds well with a threshold for low cyanobacterial abundance in shallow lakes found by Jeppesen et al. (2005) and Triest et al. (2016) and the average critical loading for shifts from turbid to clear conditions estimated for Dutch shallow lakes by Janse (2005) and in eastern England by Phillips et al. (2015). Whether external nutrient load reductions alone were responsible for the observed low in-lake TP concentrations in the lakes in our survey that reached stable clear-water conditions, however, remains questionable. It seems that in most cases additional changes in either the fish community (Lake Veluwe, Galenbecker See) and/or exotic mussel invasions (Lake Müggelsee, Eemmeer) contributed to the observed trend. In Lake Veluwe, several severe winters, an increase in bream (*Abramis brama*) fisheries between 1993 and 1997 and the increase in zebra mussel densities are all thought to have contributed to a break in the dominance of cyanobacteria, thus allowing for the prevalence of stable clear-water conditions with charophyte dominance since 1996 (Noordhuis et al., 2016, Figure 4). Once established, these dense charophyte beds provide more efficient stabilizing mechanisms for clear-water conditions than rooted angiosperms (Blindow et al., 2014). Characeae also successfully replaced *P. pectinatus* in Lake Wolderwijd after biomanipulation (Figure 4), while in Swedish Lake Krankesjön a similar development has been observed, the reasons for which are unknown (Blindow, 1992; Hargeby et al., 1994; Hansson et al., 2010). In Lake Müggelsee and Eemmeer, the additional influence of a sudden invasion of the quagga mussel (*Dreissena rostriformis bugensis*) in around 2013 might have contributed to a decline in TP concentrations and increased water transparencies (Figure 4, S. Hilt, unpublished, Noordhuis et al., 2016). This species can colonize soft substrates and thus cover much larger areas than those previously occupied by the zebra mussel (*D. polymorpha*) (Karatayev et al., 2015). In Lake Eemmeer, quagga mussels filtered the lake volume about five times a day in 2013 (Noordhuis et al., 2016).

Macrophyte recovery in Steinhuder Meer and Langer See deviates from the suggested pattern in that both are dominated by species more typical of lakes having undergone restoration with internal measures (Table 1). In Steinhuder Meer, a strong reduction of the fish population has been observed which was attributed to cormorant activities (Niedersächsischer Landesbetrieb für Wasserwirtschaft, Küsten- und Naturschutz, 2011). Cormorant effects on fish populations are also suggested for Felbrigg Lake (C. Sayer, unpublished). Through a natural increase in cormorants, the lake food web configuration was likely affected in ways comparable to those of lakes undergoing biomanipulation. This makes Steinhuder Meer and Felbrigg Lake cases of external load reduction with added unintentional internal measures (natural biomanipulation) possibly accelerating recovery. Therefore, these cases show closer correspondence to macrophyte community patterns of lakes having undergone internal measures. They also illustrate that parallel biological processes may be at play in recovering lakes that need to be considered in unison to understand the speed and trajectory of macrophyte recovery.

### Response of macrophytes to biomanipulation in shallow temperate lakes

In contrast to lakes undergoing only reduced external nutrient loading, submerged macrophytes often respond very quickly in shallow lakes subjected to biomanipulation by fish removal (Hansson et al., 1998; Bakker et al., 2013), even at rather high nutrient concentrations (Figure 2). Macrophytes colonizing these lakes are often “pioneer” species, such as *Elodea* or *Ceratophyllum*, re-colonizing from either seeds, oospores or fragments and characterized by high growth rates (Tables 2, 4). Similar species have also been recorded in lakes following natural fish kills (Sayer et al., 2016) or implementation of other in-lake restoration measures such as sediment dredging and phosphorus precipitation (Table 2). Fish removal may indirectly (due to more periphyton grazing invertebrates) reduce periphyton shading in summer, a major mechanism preventing macrophyte survival after nutrient load reduction (see Response of Macrophytes to Nutrient Load Reductions). The relevance of this process for macrophyte recovery after fish removal has not yet been directly tested, although mesocosm trials in which the density of periphyton grazers are manipulated produce predictable outcomes in terms of periphyton biomass and macrophyte composition (Elger et al., 2009). Excretion of allelopathic substances, which has been detected for many of the typical species that colonize after biomanipulation (Table 5), may also contribute to lower periphyton densities.

In general, macrophyte species typically occurring after introduction of in-lake restoration measures allow for a longer period of high macrophyte cover and dampen seasonal changes in phytoplankton abundance as described for “stable” lakes prior to major eutrophication (Sayer et al., 2010b). Both, *Elodea* and charophyte species can remain evergreen in temperate lakes (e.g., Søndergaard et al., 2017), thus extending their positive influence on water quality to seasons outside the influence of annual species.

In many cases, however, mass developments of monocultures occur. Monocultures of *Elodea* or *Ceratophyllum* species are often unstable in terms of interannual persistence (Table 5) and can collapse leading to a shift back to turbid conditions as in, for instance, Lakes Zwemlust, Væng, and Alderfen Broad (Table 2). Characeae seem less often involved in sudden collapses, although, exceptions are known, for example Schlosssee Buggenhagen (Table 2) or Lake Botshol (Rip et al., 2007). If lakes remain clear for several consecutive years, which is usually only the case at lower nutrient concentrations, a more diverse macrophyte community develops (Table 2, Lauridsen et al., 2003a). Lauridsen et al. (2003b) assumed that differences in the success of biomanipulation in Danish and Dutch shallow lakes might be attributable to variation in pioneer macrophyte species; thus, *Elodea* and *Potamogeton* species, typical for Danish lakes, were preferred over charophytes by macrophyte-grazing waterfowl (Weisner et al., 1997). Indeed, increasing top-down control of periphyton-grazing invertebrates by omnivorous fish, which increase in abundance in the period after a biomanipulation, may render macrophytes more susceptible to herbivory (Hidding et al., 2016).

### Conclusions and implications for lake management

Our analyses suggest that the composition of the macrophyte community and their seasonal abundance in shallow lakes during recovery from turbid, highly eutrophic conditions often depends on remnant macrophyte stands, the specific restoration measure applied and additional stochastic influences on water clarity such as winter fish kills, cormorant predation on fish or introduction of invasive filter-feeding mussel populations. In turn, the prevailing macrophyte community can influence lake water quality.

Reductions in external nutrient loading often result in the re-occurrence of spring clear-water phases exploitable by a few macrophyte species (mainly pondweeds) with specific traits. Resistance to wave action permits survival during the turbid phase in very shallow areas, in particular in larger lakes. During recovery these plants germinate early in spring from energy-rich vegetative propagules and complete their life cycle in early summer, when phytoplankton takes over. This intermediate recovery phase may, in some cases, last for several decades before a more diverse and abundant submerged macrophyte community develops that stabilizes clear-water conditions during the entire potential growing season (Figure 1, Table 1). Our model simulations suggest that, if the premature termination of macrophyte growth can be prevented, the summer phytoplankton peak responsible for turbid water and potentially harmful algae blooms will also be reduced. Simulations also revealed that at high periphyton shading, the intermediate recovery phase is shifted to lower nutrient loads compared with a scenario with lower periphyton shading. Therefore, if periphyton shading can be reduced external restoration measures could potentially be effective at a higher nutrient load. Macrophyte recovery during the intermediate recovery state might be facilitated by establishing exclosures to protect certain areas from herbivory by birds and/or predation of periphyton grazers by omnivorous fish, a lake-wide biomanipulation of fish, or internal measures, such as TP precipitation to lower water column TP concentrations in summer. Additional, usually unintended internal changes, such as reductions in fish abundance by commercial fisheries, natural fish kills or exotic mussel invasions can facilitate a shift to clearer conditions in summer and further aid the establishment of a more diverse macrophyte community.

In contrast, fish stock reductions, natural fish kills and sediment removal via suction-dredging can, by themselves, temporarily restore clear-water conditions in spring and summer, even at high nutrient concentrations and then allow rapid colonization by pioneer macrophyte species from *in situ* seeds, oospores or vegetative fragments. Fish stock reductions might thus be a suitable short-term management strategy, but fish removal needs to be frequently repeated.

Lasting macrophyte recovery can only be achieved in combination with reduced nutrient loading (Figure 1), a need that is further accentuated under future climate change scenarios, where cyanobacterial shading of macrophytes will likely be more severe (Kosten et al., 2012). Although global, political measures against ongoing climate warming are slow, local restoration efforts may reduce the combined stress from e.g. eutrophication and climate warming (Moss et al., 2011; Scheffer et al., 2015), and thereby serve as a buffer against further deterioration of macrophyte beds and the ecosystem services that derive from lakes and reservoirs (Urrutia-Cordero et al., 2016).

## Author contributions

SH conceived the presented idea, wrote the manuscript and performed the literature research. MA, EB, IB, TD, L-AH, EJ, TK, AK, JK, TL, RN, GP, JR, H-HS, MS, KvdW, EvD, AW, NW, and CS provided lake data. MG, JJ, AJ, WM, and ST performed the modeling. All authors contributed to discussions and the writing of different parts of the text.

### Conflict of interest statement

The authors declare that the research was conducted in the absence of any commercial or financial relationships that could be construed as a potential conflict of interest. The handling Editor is currently co-organizing a Research Topic with one of the authors EB, and confirms the absence of any other collaboration.
