# Supplementary material for: Response of Submerged Macrophyte Communities to External and Internal Restoration Measures in North Temperate Shallow Lakes
Source: Front Plant Sci. 2018 Feb 19;9:194. doi: 10.3389/fpls.2018.00194 (PMC5826081; doi:10.3389/fpls.2018.00194)
Supplement: Supplementary file 2 [file DataSheet2.DOCX]

Supplementary: Total phosphorus (TP) concentrations and Secchi depths in selected lakes (data used for Figs. 2A and B)

|  |  | Turbid phase | | | | | | Recovery phase | | | | | | Clear phase | | | | | |  |
| --- | --- | --- | --- | --- | --- | --- | --- | --- | --- | --- | --- | --- | --- | --- | --- | --- | --- | --- | --- | --- |
| No. | Lake | Data from | Mean TP Apr-Jun (mg/L) | Mean TP July-Sep (mg/L) | Secchi depth Apr-Jun (m) | Secchi depth Jul-Sep (m) | Frequency; total n | Data from | Mean TP Apr-Jun (mg/L) | Mean TP July-Sep (mg/L) | Secchi depth Apr-Jun (m) | Secchi depth Jul-Sep (m) | Frequency of data taken, period, n | Data from | Mean TP Apr-Jun (mg/L) | Mean TP July-Sep (mg/L) | Secchi depth Apr-Jun (m) | Secchi depth Jul-Sep (m) | Frequency of data taken, period, n | Data source/  contact |
| 1 | Müggelsee | 1979-89 | 0.113 | 0.248 | 1.1 | 1.1 | monthly averages of weekly measure-ments; 33 | 1990-2013 | 0.086 | 0.260 | 2.1 | 1.4 | monthly averages of weekly measure-ments; 72 | 2011-16 | 0.053 | 0.103 | 2.2 | 1.7 | monthly averages of weekly measure-ments; 18 | S. Hilt |
| 3 | Galenbecker See | 1995-2002 |  | 0.132 |  | 0.26 | once per year; 8 | 2003-06 | 0.121 | 0.228 |  | 0.1 | once per year; 4 | 2008-10 | 0.078 | 0.036 |  | 1.1 | once per year; 3 | A. Waterstraat |
| 4 | Dümmer | 1970-1999, 2003-2011 |  | 0.35 |  | 0.29 | monthly; 390 | 2012-13 | 0.12 | 0.43 | 0.8 | 0.45 | biweekly; 21 |  |  |  |  |  |  | H.H. Schuster |
| 5 | Schwielow-see | 1995, 99 |  | 0.110 |  | 0.6 | once per year; 2 | 2006, 09, 14 | 0.099 | 0.219 | 1.41 | 1.16 | monthly; 15 |  |  |  |  |  |  | T. Kabus |
| 6 | Wusterwitzer See | 1993 |  | 0.06 |  | 0.7 | monthly; 3 | 2007, 10,14 | 0.048 | 0.069 | 2.04 | 0.92 | monthly; 14 |  |  |  |  |  |  | T. Kabus |
| 7 | Grimnitzsee |  |  |  |  |  |  | 1992 | 0.042 | 0.135 | 2.4 | 0.6 | twice per year; 2 | 2008, 13, 16 | 0.032 | 0.047 | 2.17 | 1.14 | monthly; 16 | T. Kabus |
| 8 | Wardersee |  |  |  |  |  |  | 1996 | 0.15 | 0.304 | 1.45 | 1.6 | monthly; 8 |  |  |  |  |  |  | Landesamt für Natur und Umwelt Land Schles-wig-Holstein (1997) |
| 10 | Gr. Varchen-tiner See | 2006 |  | 0.161 |  | 0.2 | 1 |  |  |  |  |  |  |  |  |  |  |  |  | T. Kabus |
| 11 | Dambecker See | 2005 |  | 0.374 |  | 0.4 | 1 | 2010 | 0.097 | 0.190 | 0.8 | 0.8 | 1 |  |  |  |  |  |  | T. Kabus |
| 13 | Langer See | 1997-2001 | 0.081 | 0.131 | 0.72 | 0.38 | monthly; 30 | 2011-15 | 0.062 | 0.067 | 0.78 | 0.38 | monthly; 30 |  |  |  |  |  |  | J. Rücker |
| 14 | Gr. De Wittsee | 1992 |  | 0.490 |  | 0.4 | 1 | 2007 | 0.067 | 0.208 | 1.7 | 1.2 | 4 |  |  |  |  |  |  | K. van de Weyer |
| 16 | Felbrigg Lake | 1999-2000 | 0.072 | 0.18 | 1.29 | 0.82 | monthly; 11 | 2015-16 | 0.088 | 0.317 |  |  | monthly; 14 |  |  |  |  |  |  | C. Sayer |
| 17 | Barton Broad | 1974-90 | 0.212 | 0.24 | 0.51 | 0.4 | monthly; 102 | 1990-2000 | 0.085 | 0.157 | 0.64 | 0.42 | monthly; 66 |  |  |  |  |  |  | G. Phillips, A. Kelly |
| 18 | Wolderwijd | 1975 |  |  | 0.45 | 0.28 | biweekly, 11 | 1982-90 | 0.146 | 0.162 | 0.39 | 0.30 | biweekly; 112 |  |  |  |  |  |  | E. van Donk, R. Noordhuis |
| 19 | Veluwemeer | 1975 | 0.5 | 0.694 | 0.27 | 0.27 | biweekly; 12 | 1976-95 | 0.094 | 0.131 | 0.33 | 0.26 | yearly means of biweekly measure-ments; 20 | 1996-2016 | 0.048 | 0.051 | 0.82 | 0.73 | yearly means of biweekly measure-ments; 21 | R. Noordhuis |
| 20 | Eemmeer | 1971-99 | 0.709 | 0.917 | 0.25 | 0.23 | yearly means of biweekly measure-ments; 28 | 2000-16 | 0.153 | 0.221 | 0.69 | 0.57 | yearly means of biweekly measure-ments; 28 |  |  |  |  |  |  | R. Noordhuis |
| 21 | Arreso | 1989-95 | 0.355 |  |  | 0.4 | range for all years; ? |  |  |  |  |  |  |  |  |  |  |  |  | M. Sondergaard |
| 22 | Duiniger-meer | 1991-92 | 0.072 | 0.125 | 0.57 | 0.48 | biweekly; 12 | 1995-96 | 0.069 | 0.049 | 0.92 | 1.08 | biwekly; 18 |  |  |  |  |  |  | E. van Donk |
| 23 | Ijzeren Man | 1986-89 | 0.16 | 0.298 | 0.29 | 0.21 | monthly; 22 | 1990-94 | 0.121 | 0.155 | 0.49 | 0.5 | monthly, 24 |  |  |  |  |  |  | E. van Donk |
| 24 | Noorddiep | 1987 | 0.21 | 0.215 | 0.29 | 0.34 | biweekly; 12 | 1989-95 | 0.403 | 0.59 | 0.96 | 0.79 | biweekly-monthly, 54 |  |  |  |  |  |  | E. van Donk |
| 25 | Wolderwijd |  |  |  |  |  |  | 1994-99 | 0.078 | 0.077 | 0.61 | 0.62 | biweekly-monthly; 52 |  |  |  |  |  |  | E. van Donk, R. Noordhuis |
| 26 | Zwemlust 1987 | 1986 | 1 | 0.95 | 0.23 | 0.23 | biweekly, 10 | 1988-96 | 0.844 | 0.828 | 1.99 | 1.68 | biweekly; 125 |  |  |  |  |  |  | E. van Donk |
|  | Zwemlust 1999 | 1997-98 | 0.439 | 0.806 | 1.1 | 0.69 | biweekly; 21 | 2000 | 0.265 |  | 2.42 | 2.2 | biweekly; 11 |  |  |  |  |  |  | E. van Donk |
| 34 | Vaeng 1986 | 1981-86 |  | 0.13 |  | 0.6 | summer means; monthly | 1987-96 |  | 0.11 |  | 1.4 | average of summer means; 10 |  |  |  |  |  |  | from Sondergaard et al. (2017) |
|  | Vaeng 2007 | 2006-07 |  | 0.13 |  | 0.7 | average of summer means; 2 | 2010-15 |  | 0.05 |  | 1.6 | average of summer means; 6 |  |  |  |  |  |  | from Sondergaard et al. (2017) |
| 35 | Arreskov | 1991 |  | 0.25 |  | 0.3 | ? |  |  |  |  |  |  |  |  |  |  |  |  | M. Sondergaard |
| 36 | Alderfen | 1974-80 | 0.185 | 0.297 |  |  | monthly; 42 | 2000-12 | 0.081 | 0.078 | 1.07 | 0.98 | monthly; 78 |  |  |  |  |  |  | G. Phillips, A. Kelly |
| 37 | Cockshoot Broad | 1970-80 | 0.16 | 0.15 |  |  | monthly; 66 | 1990-2000 | 0.064 | 0.121 | 0.89 | 0.9 | monthly; 66 |  |  |  |  |  |  | G. Phillips, A. Kelly |
| 38 | Hoveton Little Broad | 1983-89 | 0.111 | 0.111 |  |  | monthly; 42 | 1990-2000 | 0.081 | 0.153 | 1.35 | 0.99 | monthly; 66 |  |  |  |  |  |  | G. Phillips, A. Kelly |
| 39 | Ormesby Great Broad | 1970-89 | 0.049 | 0.061 | 1.15 | 0.8 | monthly; 120 | 1995-2010 | 0.047 | 0.087 | 0.86 | 0.81 | monthly; 96 |  |  |  |  |  |  | G. Phillips, A. Kelly |
| 40 | Cromes | 1970-90 | 0.15 | 0.111 |  |  | monthly; 126 | 1991-2012 | 0.081 | 0.153 | 0.83 | 0.83 | monthly; 132 |  |  |  |  |  |  | G. Phillips, A. Kelly |
| 41 | Barton Broad |  |  |  |  |  |  | 2000-2012 | 0.049 | 0.083 | 1.02 | 0.69 | monthly; 78 |  |  |  |  |  |  | G. Phillips, A. Kelly |
| 42 | Schollener See | 1993-2002 |  | 0.257 | 0.51 | 0.4 | yearly means (Apr-Oct); 6 | 2004 |  | 0.15 |  | 1 | monthly, 4 |  |  |  |  |  |  | from Knösche (2008) |
| 44 | Schwandter See | 1995, 99, 2001 | 0.085 | 0.142 |  | 0.33 | spring/ summer mean; ? | 2002-12 | 0.057 | 0.073 | 1.12 | 1.48 | spring/ summer mean; ? |  |  |  |  |  |  | from Nixdorf et al. (2013) |
| 45 | Ivenacker See | 2003 | 0.125 | 0.117 | 0.32 | 0.29 | monthly; 6 | 2010 | 0.105 | 0.103 |  |  | monthly; 6 |  |  |  |  |  |  | from Nixdorf et al. (2013) |
| 46 | Schlosssee Buggenhagen | 1996-97 |  | 0.199 |  |  | summer mean; ? | 1998-99 |  | 0.038 |  |  | summer mean; ? |  |  |  |  |  |  | from Mathes (2007) |
